# Evaluation of clinical, histology, TNF-α, and collagen expressions on oral ulcer in rats after treatment with areca nut and chrysanthemum oral gel

**DOI:** 10.12688/f1000research.54887.2

**Published:** 2021-10-19

**Authors:** Liza Meutia Sari, Zaki Mubarak, Dina Keumala Sari

**Affiliations:** 1 Oral Medicine, Syiah Kuala University, Banda Aceh, Indonesia, 23111, Indonesia; 2 Oral Microbiology, Syiah Kuala University, Banda Aceh, Indonesia, 23111, Indonesia; 3 Nutrition, Sumatera Utara University, Medan, Indonesia, 20155, Indonesia

**Keywords:** Oral ulcer, Areca nut, Chrysanthemum, TNF-α, Collagen

## Abstract

**Background: **Areca nut ( *Areca catechu* Linn.) is the seed of the fruit of the oriental palm that is commonly used among Southeast Asian communities. Chrysanthemum ( *Dendrathema grandiflora*) is a flowering plant originating from East Asia and dominantly grows in China. Both of these plants have strong antioxidant activities. To investigate the mechanism of their wound healing activities, we prepared areca nut and chrysanthemum polyethylene oral gel and performed several *in vivo* assays using Sprague–Dawley rats.

**Methods: **Sprague–Dawley rats were divided into five groups: Negative control group (rats with base gel treatment), positive control group (rats treated with triamcinolone acetonide), F1 (treatment with 20% areca nut:80% chrysanthemum), F2 (treatment with 50% areca nut:50% chrysanthemum), and F3 (treatment with 80% areca nut:20% chrysanthemum). Traumatic ulcers were performed on the buccal mucosa of all experimental animals that received topical oral gel and triamcinolone acetonide twice a day for seven days. The clinical and histological characteristics were analyzed and scored.

**Results:** During the six days, the ulcerated area receded linearly over time and was completely cicatrized in F2, F3, and positive control group (Dependent t-test, p<0.05). There were significant increases in body weight in F2 and positive control groups. There were no significant differences between groups in histology examination (Kruskal Wallis test, p<0.05). The moderate score of TNF-α levels was seen in F2 and positive control groups (ANOVA/Tukey test, p<0.05). In the collagenase assay, high concentration of areca nut (F3) induced the abundant of collagen during the ulcer healing process.

**Conclusions: **A combination of areca nut and chrysanthemum extract in the oral gel can optimize the healing of traumatic oral ulcers in Sprague-Dawley rats through the increase of TNF-α and collagen deposition.

## Introduction

Oral ulceration is the most common presentation in the oral cavity caused by many etiologic factors. ^1^ Most of the etiologies of ulcerative lesions on the oral mucosa are divided into four categories; namely, infectious (bacterial and virus infection), immune-related (autoimmune and allergic), traumatic (mechanical trauma), and neoplastic (oral squamous cell carcinoma). ^2^ Traumatic ulcers are injuries to the oral mucosa caused by mechanical or physical trauma such as sharp food, accidental biting during mastication, biting while speaking, punctured by sharp objects, fractured, malformed, carious, or malposed teeth on the superficial epithelial layer or underlying connective tissue or may involve both. ^3^

Currently, increasing the resolution of wound healing is one of the main priorities in the medical field to accelerate the healing of chronic wounds and traumatic injuries. ^4^ The process of tissue regeneration and repair occurs immediately after lesion onset. The linear tissue repair and regeneration involves growth factors that induce cell proliferation, especially parenchymal cells, followed by dynamic changes in soluble mediators, blood cells, and the extracellular matrix. ^5^ The unique oral cavity environment shows advantages in accelerating wound healing compared with skin repair. This is due to differences in the response to inflammation, differentiation and proliferative programs, modulation of stem cells, collagen synthesis, the role of macrophages, and epithelial remodeling. ^6^ ^,^ ^7^ Collagen synthesis and tumor necrosis factor-α (TNF-α) are important components during acute inflammation and responsible for a diverse range of signaling events within cells in the wound-healing process. TNF-α is one of inflammatory cytokine and regulates the immune system. ^8^

Areca nut is the seed of the areca palm ( *Areca catechu* Linn.; *areca, Palmaceae*), which grows thrive in tropical Pacific region, South and South East Asia, including Indonesia where it is known as “ *Pinang*”. It has been consumed by people worldwide as part of ancient tradition, custom, or ritual for a long time. Several studies have been conducted to prove the healing ability of areca nut, including that the alkaloid and polyphenols content in areca nut could enhance the healing of burn wounds, leg ulcers, and skin graft surgery. ^9^ ^,^ ^10^ Areca nut contains alkaloid, phenolic, tannin, and flavonoid compounds. The presence of catechin, quercetin, and jacareubin contributed antioxidant and cytotoxic activities of areca nut extract. ^1^ Our previous study showed that areca nut extract had cytotoxic activity againts human oral cancer cell lines such as HSC-2 and HSC-3 with IC _50_ values of 629.50 and 164.06 μg/mL, respectively. ^2^ The selectivity of the areca nut extract was verified by using normal cell line. Areca nut had no cytotoxic effect on human keratinocyte (HaCaT) cell line. Instead, it induced high rate of proliferation in HaCaT cells. Chrysanthemum or chrysanth ( *Dendrathema grandiflora; Asteracea*) is a flowering plant originating from East Asia and dominantly grows in China. The flowers are generally consumed in herbal teas and as a supplement. This genus comprises 40 species and contains flavonoids (flavanone), terpenoids, anthocyanins, and steroids. ^3^ ^,^ ^4^ The selectivity index of *Chrysanthemum indicum* and *morifilium* oils were calculated 1.8 and 1.5, respectively, indicating low selectivity against trypanosomes compared to their toxicity versus HL-60 cells. ^5^ Previous studies reported the effect of increasing keratinocyte proliferation and skin regeneration derived from *Chrysanthemum boreale.* ^11^ Chrysanthemums are usually used for treating allergies, anxiety, hypertension, inflammation, headache, cold, sore throat, and tinnitus by certain communities. ^12^

However, scientific research has not been conducted related to the healing process on the oral mucosa by oral application of these herbal plants. This study aimed to evaluate the potential of the combination of areca nut and chrysanthemum oral gel on oral mucosa using the Sprague–Dawley rat model.

## Methods

### Ethical approval

This study was approved by the Ethics Committee for Animal Research of Tropical Biopharmaceutical Research Center (Trop BRC), Bogor Agricultural University, West Java, Indonesia, with number 042-2020-KEH TROP BRC.

### Extract preparation

Areca nuts were obtained from the *Pinang* plant from Aceh Besar, Indonesia, which was documented and determined by the Botanical Division of Biological Research Center LIPI Cibinong, complete with its roots, stems, leaves, flowers, and seeds in 2017. 2 kg of areca nuts (gross weight) were cleansed of dirt and dried in the open air and sunlight. Further drying was done using an oven set at a temperature of 50°C. The nuts were crushed using a blender and then strained with a 20-mesh sieve. The maceration process was conducted using 96% ethanol diluent solvent for 7 days before being subsequently filtered and evaporated using a vacuum rotary evaporator at 30–40°C and then reconcentrated using a water bath until a solid dry powder extract was obtained. Chrysanthemum polyethylene (P.E.) (Product No. 1237X17911) was provided by Javaplant Company, Indonesia.

### Preparation of areca nut and chrysanthemum extract oral gel

The composition of the optimum formula was determined from 3 concentrations of carboxymethyl cellulose-Natrium (CMC-Na), followed by an evaluation of the physical stability study. The formula included in the criteria parameters is selected as the optimum formula. The compositions of the formula are shown in Table 1. The gels were made by dissolving areca nut and chrysanthemum extract with distilled water and then heated at a temperature of 50°C. The CMC-Na was heated with the remaining distilled water on a magnetic stirrer with a stirring speed of 400 rpm at a temperature of 70°C. Methylparaben was added until dissolved. Propylene glycol and glycerin were mixed and then added to the mixture of CMC-Na and methylparaben. The liquefied extract was added to the mixture and stirred continuously until a gel was formed.

**Table 1.** The compositions of areca nut and chrysanthemum oral gel formula.

Ingredient	Concentration (%)			Function
	F I	F II	F III	
Areca nut extract	5	5	5	Active substance
Chrysanthemum extract	5	5	5	Active substance
CMC-Na	3	4	5	Gelling agent
Propylene glycol	15	15	15	Humectant
Glycerin	10	10	10	Humectant
Methylparaben	0.25	0.25	0.25	Preservative
Distilled water ad	100	100	100	Solvent

Formulation I: 3% CMC-Na, Formulation II: 4% CMC-Na, Formulation III: 5% CMC-Na.

### Optimization of areca nut and chrysanthemum oral gel formula

The prepared formulations were placed in an incubator at a temperature of 40°C for 4 weeks. After that period time, all formulations were placed in an incubator at 5°C for optimum formulation selection. The final results are shown in Table 2. Formulation F III was found to be the most stable after the preliminary study. This optimum formula was used for the next assays.

**Table 2.** Characteristics of areca nut and chrysanthemum oral gel formula.

Evaluation	Criteria parameter	Characteristics		
		F I	F II	F III
Homogeneity	Homogeneous	Inhomogeneous	Homogeneous *	Homogeneous *
Consistency	Soft viscous	Liquid	Liquid	Soft viscous *
pH	4.5-6.5	5.72 ± 0.02 *	5.88 ± 0.15 *	6.02 ± 0.07 *
Spreadability (cm)	5-7	7.05 ± 0.32	7.02 ± 0.22	5.5 ± 0.34 *

Formulation I: 3% CMC-Na, Formulation II: 4% CMC-Na, Formulation III: 5% CMC-Na.

* In accordance with the parameters.

### Physical stability study

The optimum formula of the areca nut and chrysanthemum P. E oral gel (F III) was evaluated for thermal stability by observing color, shape, odor, taste, homogeneity, pH, spreadability, and viscosity during storage with a constant relative humidity (RH) of 75 ± 5% and maintained at 40 ± 2°C, without direct light. The samples were analyzed at the initial time (t _0_) and 2, 4, 6, and 8 weeks after exposure to the atmospheric conditions. **The homogeneity test** was carried out by visual inspection after the gels have been set in the container. ^6^ They were tested for their appearance and presence of any aggregates. **The pH test** is carried out to observe the acidity level of the gel preparation to ensure that the oral gel does not irritate the skin. The pH criteria of oral gel are in the interval 4.5-6.5. ^7^ **The spreadability test** was carried out to ensure the even distribution of the gel when applied to the skin. ^8^ The gel was weighed as much as 0.5 grams and then placed in the middle of a round glass scale. On top of the gel is placed another round glass and ballast so that the weight of the round glass and ballast is 150 grams, allowed to stand for 1 minute, then the diameter of the distribution is analyzed. The good spreadability is between 5-7 cm. **The viscosity test** was analyzed by putting the gels in the Brookfield Viscometer and was rotated at 50 rpm using spindle no. 64. The corresponding dial reading was noted. ^6^

### Formulation of areca nut and chrysanthemum extract oral gel

The extract was formulated into four gel dosage forms with various concentrations of the extract combination, namely F1 (20% of areca nut:80% of chrysanthemum), F2 (50% of areca nut:50% of chrysanthemum), F3 (80% of areca nut:20% of chrysanthemum), and base gel (gel without areca nut and chrysanthemum). Preparation of the extract oral gel began with making two mixtures. The first mixture consisted of a carbamoyl and extract combination of areca nut and chrysanthemum and mixed with 10 mL of water at 70°C. The second mixture consisted of methylparaben dissolved in a little water and then mixed with a mixture of glycerin and propylene glycol. This mixture was then combined and stirred with water so that it is mixed homogeneously.

### Experimental animals

A total of 25 adult male Sprague–Dawley rats weighing 200–240 g were provided by the animal laboratory at Tropical Biopharmaceutical Research Center (Trop BRC), Bogor Agricultural University. The rats were kept for adaptation at 25°C in the well-ventilated laboratory for 14 days under a 12/12 h light/dark cycle and fed with a standard pellet diet and tap water *ad libitum* before being entered into the experiment. All the animals were given an initial examination for systemic health conditions and stored in boxes with sawdust.

### Experimental protocol to induce the ulcers

Ulcer induction was performed after the animals were anesthetized by intraperitoneal injection of 33 mg/kg of ketamine and 13 mg/kg of xylazine (2%). The left buccal mucosa was smeared with 10% povidone–iodine on cotton pellets. An abrasion was made with 5 mm diameter, 1 mm depth, and was limited to the mucosa without muscular involvement. The ulceration was performed by using a number 15 scalpel blade. The standardization area was marked with an 8-mm diameter demarcator. The surgical and technique was standardized for animals and performed by the same operator. ^13^ The ulceration was not performed in the normal group. The formation of the ulcers could be observed after 24 hours.

### Groups and treatment

The animals were randomly divided into five groups with 12 animals each: negative control group (rats with base gel treatment), positive control group (rats treated with triamcinolone acetonide), F1 (treatment with 20% areca nut:80% chrysanthemum), F2 (treatment with 50% areca nut:50% chrysanthemum), and F3 (treatment with 80% areca nut:20% chrysanthemum). A normal group (without ulcer and treatment) was added for the microscopic evaluation. All the groups were treated every 12 hours for seven days with topical application. The application of the oral gel was performed by using the individual sterile and disposable dental micro brush (SDent, USA). Each group was sacrificed gradually through the end of the seventh-day study period. After the animals were sacrificed, a section of buccal mucosa containing the ulcer of each rat was collected. Histopathology, immunohistochemistry, and collagenases evaluation were analyzed in Primate Research Center, Bogor Agricultural University, Indonesia.

### Clinical evaluation

The animal body weights and diameter of the ulcers were measured before and after treatment on the second and seventh days. The diameter of the ulcers were measured with the naked eyes using a digital stainless vernier caliper which is capable of measuring diameter in 0.05 mm increments. ^14^ ^,^ ^15^ All measurements were performed by the same operator.

### Histopathological analysis

The collected fragments of ulcers were identified and immersed in 10% formol for 24 hours. After fixation in formol, the specimens were macroscopically analyzed, subjected to dehydration in crescent alcoholic series, diaphanized in xylol, impregnated in paraffin, and melted at 60°C. The fragments were packed in paraffin-forming blocks at room temperature. The fragments were sectioned to 5 μm in thickness through the use of microtome and histology using routine coloration by hematoxylin-eosin (HE) was performed. The histopathological parameters were determined and scored from 0 to 4 according to previously published criteria ( Table 3). ^16^

**Table 3.** Histopathologic analysis of induced oral ulcers (histological scores).

Score	Epithelium	Conjunctive tissue
0	No ulcer	Remodeled
1	No ulcer	Fibrosis and slight chronic inflammation
2	With ulcer	Fibrosis and moderate chronic inflammation
3	With ulcer	Chronic inflammation process (granulation tissue)
4	With ulcer	Acute process (dilated vessels, mixed inflammatory infiltrate with neutrophils)

### Immunohistochemistry analysis

The selected tissue sections of the ulcers (2.5 μm) were deparaffinized and rehydrated with distilled water. Blocking endogen peroxidase activity was done using 3% hydrogen peroxide solution for 15 minutes, followed by washing with phosphate-buffered saline (PBS) three times every 5 minutes. After protein blocking, the dripping of Biocare’s Background Sniper was performed and the specimens were incubated for 15 minutes at 37°C. The dripping of normal serum was performed and incubated for 60 minutes at 37°C. The specimens were washed with PBS and anti-TNF-α antibody (RRID: AB_2892586; TNFA/1172) (ab220210Abcam), was diluted at 1:100, and then dripped and incubated at 4°C for two days. After washing with PBS, the secondary antibody goat anti-rabbit IgG H&L horseradish peroxidase (HRP) (RRID: AB_955447; ab6721-Abcam) was given for 30 minutes at 25°C and followed by Betazoid DAB chromogen solution (BDB2004H-Biocare Medicare LLC). It was used for increasing stability and staining intensity for HRP detection in the specimens. The specimens were washed with distilled water and checked with the microscope. After counterstaining with hematoxylin for 30 seconds, the specimens were rinsed in tap water, dehydrated, purified, and mounted. The percentage of nuclear and cytoplasmic expression in the connective tissue was divided into four scores, namely: 0: no positive cells; 1 (mild): 1–33% of positive cells; 2 (moderate): 34–66% of positive cells; 3 (intense): 67–100% of positive cells. Two observers analyzed the same scores until they were considered as the final scores. ^17^

### Collagenase analysis

The same fragments were sectioned to a thickness of 3 μm, de-wax, hydrate paraffin section, and stained using picrosirius red (solution A) for one hour. After washing in acidified water (solution B), the specimens were dehydrated and mounted for observation under polarized light microscopy. The collagen bundles shown in red in the image were then calculated using Adobe Photoshop CC 2017 (RRID:SCR_014199; GNU Image Manipulation Program (RRID:SCR_003182) is an open-access alternative) and ImageJ (v1.50i) (RRID:SCR_003070) software. The percentage of collagen was derived from collagen area pixel divided by tissue pixel and multiply by 100%. The mean of three percentages was used as a sample unit. ^18^

### Statistical analysis

All experiments were carried out in triplicate and the data were expressed as mean ± standard deviation (SD). Statistical analysis was performed by using the SPSS 20 software (SPSS Inc., Chicago, USA) ( **RRID:SCR_019096**); JASP ( **RRID:SCR_015823**) is an open-access alternative. The spreadability and viscosity test analyzed by one-way analysis of variance (ANOVA/Tukey test). Clinical evaluation in changes of the area of ulcer and body weight of rats analyzed by the dependent t-test. Differences among samples in histopathological and immunohistochemistry analysis were evaluated by using the Kruskal–Wallis test. Collagenesis analysis was evaluated by using ANOVA/Tukey test. A significant difference was assumed at *p* < 0.05.

## Results

### Physical stability evaluation

The selected formulation F III presented appreciable organoleptic properties during stability studies. The color, smell, taste, and homogeneity didn’t show significant changes, but a slight changes in shape and pH were seen in the 8 ^th^ week ( Table 5). Data of spreadability test in optimum formula gel showed that the longer the storage time, the gel will spread wider. One-Way ANOVA analysis showed that there was a significant difference in the average dispersion of the gel every 2 weeks, between week 0 with week 4, 6, and 8 (mean = 5.53 ± 0.09, 95% CI: [−1.37, −7.36]), so it can be concluded that this gel formula was unstable in spreading ability during 8 weeks storage in 40°C. Different results are found in the viscosity test. The viscosity of the gel showed the reduction of viscosity value every 2 weeks of storage time, but the difference was not significant ( *p* > 0.05) These results can be seen in Table 5.

### Clinical evaluation

**Figure 1.**
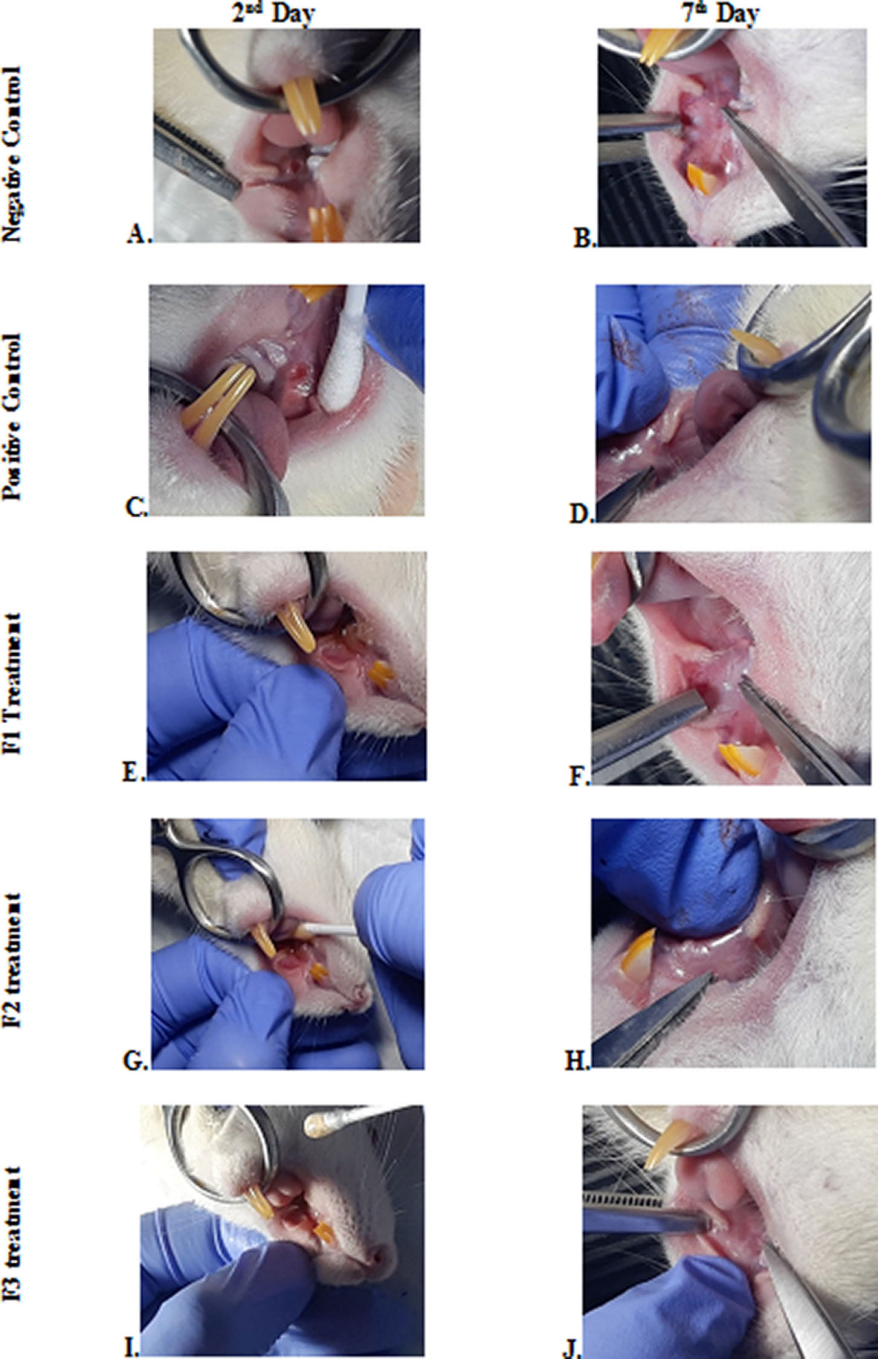
Clinical analysis of the traumatic ulcers in the buccal mucosa on the second and seventh day. Negative control: base gel treatment group (A and B); Positive control: triamcinolone acetonide treatment (C and D); F1 treatment: 20% areca nut:80% chrysanthemum (E and F); F2 treatment: 50% areca nut:50% chrysanthemum (G and H); F3 treatment: 80% areca nut:20% chrysanthemum (I and J).

The change comparison of ulcer size analysis in the buccal mucosa of rats showed a significant decrease in ulcer size in F1, F2, F3, and positive control groups ( Figure 1A–J, Table 4). This observation was carried out on the second day after the ulcer was formed and at the end of the experiment. The most significant decrease in ulcer area was seen in positive control (triamcinolone acetonide orabase), F2, and F3 treatment groups. Notably, the average ulcer size in positive control, F2, and F3 treatment on the second day was 10.10 ± 0.17, 10.08 ± 0.16, and 10.08 ± 0.18 mm ^2^, respectively. On the seventh day, it was seen that the size of the ulcer had almost closed, namely 0.03 ± 0.01, 0.11 ± 0.07, and 3.49 ± 0.42 mm ^2^, respectively. In the end of experimental period, the clinical evaluation showed that although ulcers in the F2 group has smaller size, but the ulcers in the F3 group have tighter healing wound closure and more cicatricial tissue formation than other treatment groups. These data analysis showed that treatment with areca nut and chrysanthemum oral gel caused significantly ( *p* < 0.05) ulcer healing in a dose-dependent manner in clinical analysis. Analysis of changes in bodyweight showed a significant increase in the F2 and positive control groups, while a significant decrease in bodyweight occurred in the F1 and F3 treatment groups ( Table 4). Meanwhile, in the negative control group, there was a decrease in bodyweight, but it was not significant.

**Table 4.** Effect of areca nut and chrysanthemum oral gel on the area of ulcer and body weight changes in rats on the 2nd and 7th experimental days (n = 60).

Groups	Clinical evaluation							
	Area of ulcer changes (mm ^2^)				Body weight changes (g)			
	2 ^nd^ day	7 ^th^ day	95% CI	*p*	2 ^nd^ day	7 ^th^ day	95% CI	*p*
Negative control	9.34 ± 0.69	5.24 ± 8.89	6.34, 6.84	0.000	118.58 ± 5.29	117.25 ± 6.19	−1.31, 3.97	0.291
Positive control	10.10 ± 0.17	0.03 ± 0,01	9.96, 10.19	0.000	109.58 ± 6.89	117.17 ± 7.50	−12.31, −2.84	0.005
F1	10.04 ± 0.18	2.84 ± 0.44	6.89, 7.52	0.000	119.17 ± 13.27	115.33 ± 14.46	2.12, 5.54	0.000
F2	10.08 ± 0.16	0.11 ± 0.07	9.86, 10.08	0.000	116.83 ± 4.41	121.58 ± 4.87	−8.10, −1.39	0.010
F3	10.08 ± 0.18	1.81 ± 0.71	6.25, 7.59	0.000	120.50 ± 4.32	117.75 ± 6.82	0.35, 5.14	0.028

Data are expressed as the mean ± SD, 95% CI, *p* < 0.05 dependent t-test, Results are representative of three independent experiments. Negative control: base gel treatment group; Positive control: triamcinolone acetonide treatment; F1: 20% areca nut:80% chrysanthemum; F2: 50% areca nut:50% chrysanthemum; F3: 80% areca nut:20% chrysanthemum.

**Table 5.** Organoleptics characteristics and physical stability tests of F III kept at 40°C/75% RH.

Evaluation	Weeks of storage						
	0	2	4	6	8	95% CI	*p*
**Organoleptic characteristics**							
**Color**	Dark chocolate	Dark chocolate	Dark chocolate	Dark chocolate	Dark chocolate		
**Aroma**	Typical aromatic	Typical aromatic	Typical aromatic	Typical aromatic	Typical aromatic		
**Taste**	Bitter	Bitter	Bitter	Bitter	Bitter		
**Shape**	Condensed	Condensed	Liquid	Liquid	Liquid		
**Homogeneity**	Homogeneous	Homogeneous	Homogeneous	Homogeneous	Homogeneous		
**pH**	5.50 ± 0.03	5.52 ± 0.02	5.50 ± 0.03	5.45 ± 0.02	4.49 ± 0.01		
**Spreadability (cm)**	5.53 ^a^ ± 0.09	5.72 ^a^ ± 0.08	6.21 ^b^ ± 0.18	6.73 ^c^ ± 0.15	6.79 ^c^ ± 0.09	−1.37, −7.36 ^d^	0.000
**Viscosity (m.Pas)**	202.24 ^e^ ± 1.12	184.52 ^e^ ± 4.67	181.03 ^e^ ± 0.98	161.96 ^e^ ± 2.73	155.82 ^e^ ± 0.45	−4.54, 35.42 ^d^	0.129

Data are expressed as the mean ± SD, 95% CI, *p* < 0.05, One-Way ANOVA/Tukey Post Hoc test. Results are representative of three independent experiments.

^a^ Significantly different from the week 4, 6, and 8;

^b^ Significantly different from the week 0, 2, 6, and 8;

^c^ Significantly different from the week 0, 2, and 4;

^d^ 95% CI week 0 vs week 8,

^e^ No significant differences of viscosity among weeks of storage time ( *p* > 0.05).

**Table 6.** Effect of areca nut and chrysanthemum oral gel on histological scores, number of connective tissue cells that expressed TNF-α, and percentage of collagen deposition area on the 7 ^th^ experimental day (n = 18).

Parameter	Microscopic evaluation							
	Groups							
	Negative control	Positive control	F1	F2	F3	Normal	95% CI	*p*
Histological scores	0	1.34 ± 1.53	1.67 ± 1.53	0	1.34 ± 0.58	0		0.107
TNF-α immunostaining scores	1.33 ± 0.58	2.67 ± 0.58	1.00 ± 1.53	2.33 ± 0.58	1.33 ± 0.58	1.00 ± 0		0.025
Collagen deposition area (%)	5.63 ^a^ ± 2.28	14.49 ± 0.95	17.73 ^b^ ± 9.32	12.63 ^c^ ± 0.92	25.4 ^d^ ± 2.12	9.40 ± 1.05	1.61, 23.93 ^e^	0.001

Data of histological and TNF-α immunostaining scores are expressed as the mean±SD, *p* < 0.05, Kruskal-Wallis test. Data of collagenase assay are expressed as the mean ± SD, 95% CI, *p* < 0.05, One-Way ANOVA/Tukey Post Hoc test. Results are representative of three independent experiments.

^a^ Significantly different from F1 and F3 groups.

^b^ Significantly different from negative control.

^c^ Significantly different from F3.

^d^ Significantly different from F2, negative control, and normal groups.

^e^ 95% CI of F2 compared with F3. Negative control: base gel treatment group; Positive control: triamcinolone acetonide treatment; F1: 20% areca nut:80% chrysanthemum; F2: 50% areca nut:50% chrysanthemum; F3: 80% areca nut:20% chrysanthemum.

### Histological evaluation

**Figure 2.**
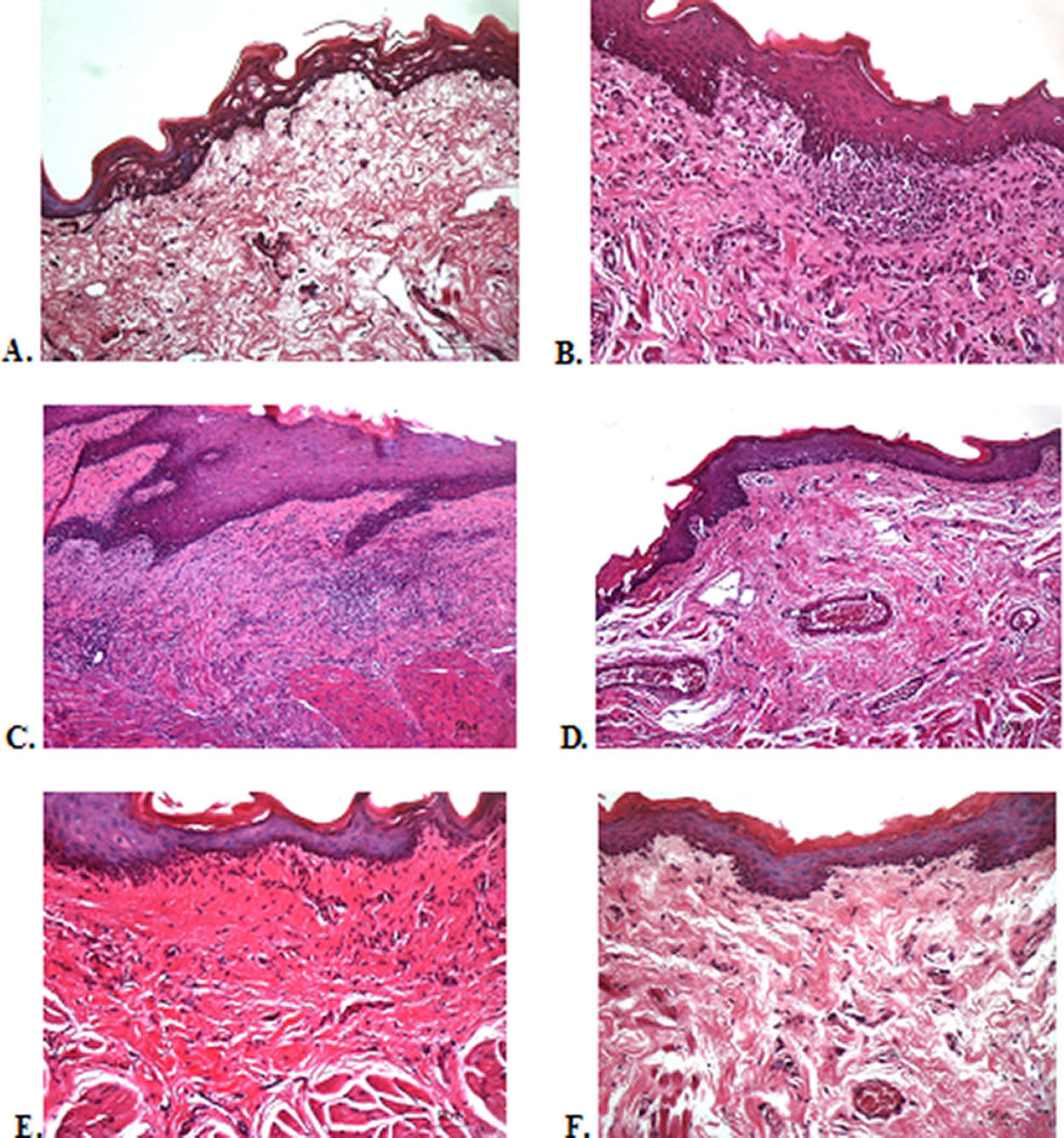
Histopathological analysis of the traumatic ulcers in the buccal mucosa at second and seventh day. (A) Negative control: base gel; (B) Positive control: triamcinolone acetonide in orabase; (C) F1: 20% areca nut:80% chrysanthemum; (D) F2: 50% areca nut:50% chrysanthemum; (E) F3: 80% areca nut:20% chrysanthemum; (F) Normal group: without ulcer and treatment (Hematoxylin and eosin, 40×).

To verify the mechanism of ulcer healing by areca nut and chrysanthemum oral gel, the ulcers were subjected to histological examination. As seen in Table 6, the mean of histological scores on the seventh day showed that positive control, F1, and F3 groups indicated that there were no ulcers in the epithelial layer, presence of fibrosis, and slight chronic inflammation in the conjunctive tissue. The score 0 indicating no ulcers and remodeling in the conjunctive tissue was seen in the negative control, F2, and normal groups, but significant differences between groups were not detected in histology examination. Analysis of photomicrograph of ulceration showed loss of ulcer with remodeled connective tissue and accompanied by a slight inflammatory infiltrate in Figure 2B, 2C, and 2E (score 1). There were no visible inflammations in Figure 2A, 2D, and 2F (score 0).

### Immunohistochemical evaluation

**Figure 3.**
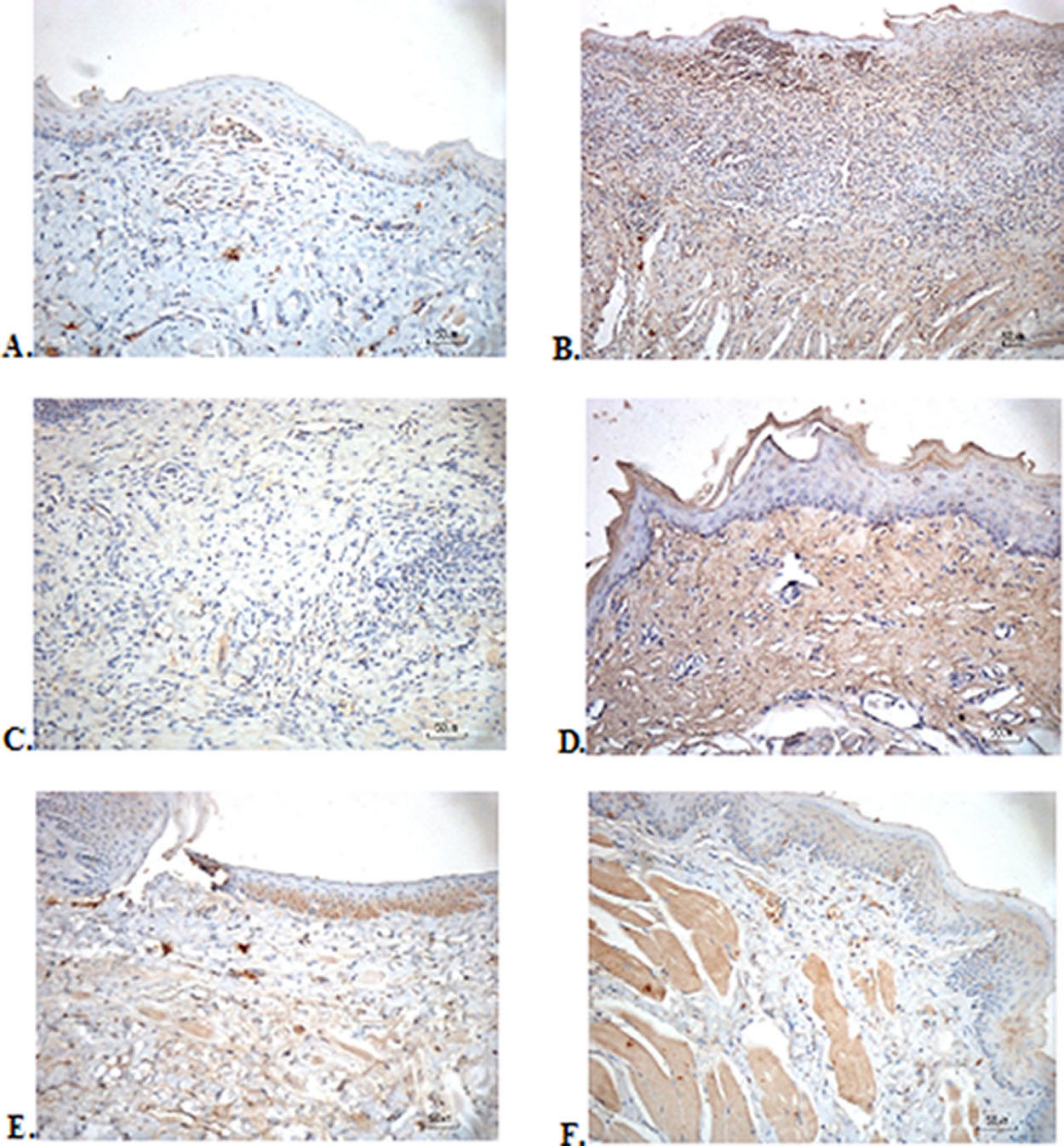
Immunohistochemistry analysis of the traumatic ulcers in the buccal mucosa on the second and seventh day. (A) Negative control: base gel; (B) Positive control: triamcinolone acetonide in orabase; (C) F1: 20% areca nut:80% and chrysanthemum; (D) F2: 50% areca nut:50% chrysanthemum; (E) F3: 80% areca nut:20% chrysanthemum; (F) Normal group: without ulcer and treatment (TNF-α immunostaining, 40×)

The immunostaining for TNF-α aims to show changes in TNF-α expression released by connective tissue cells in the cytoplasm during the healing process ( Figure 3). The increase in the expressions with a mean score of 2 (moderate) was showed by positive control (2.67 ± 0.58) and F2 groups (2.33 ± 0.58) ( Table 6). The lower scores (1-mild) were seen in the negative control, F1, F3, and normal groups. This study showed significant differences between the six groups with changes in TNF-α expression. These results were in line with the pictures that revealed brown diffuse granular-like staining pattern of the connective tissue and epidermis in the Figure 3B and 3D, while the other groups did not show dominant TNF-α expressions except in the F3 group, the immunostaining of TNF-α was prominently found in the basal cell layer ( Figure 3E).

### Collagenase evaluation

**Figure 4.**
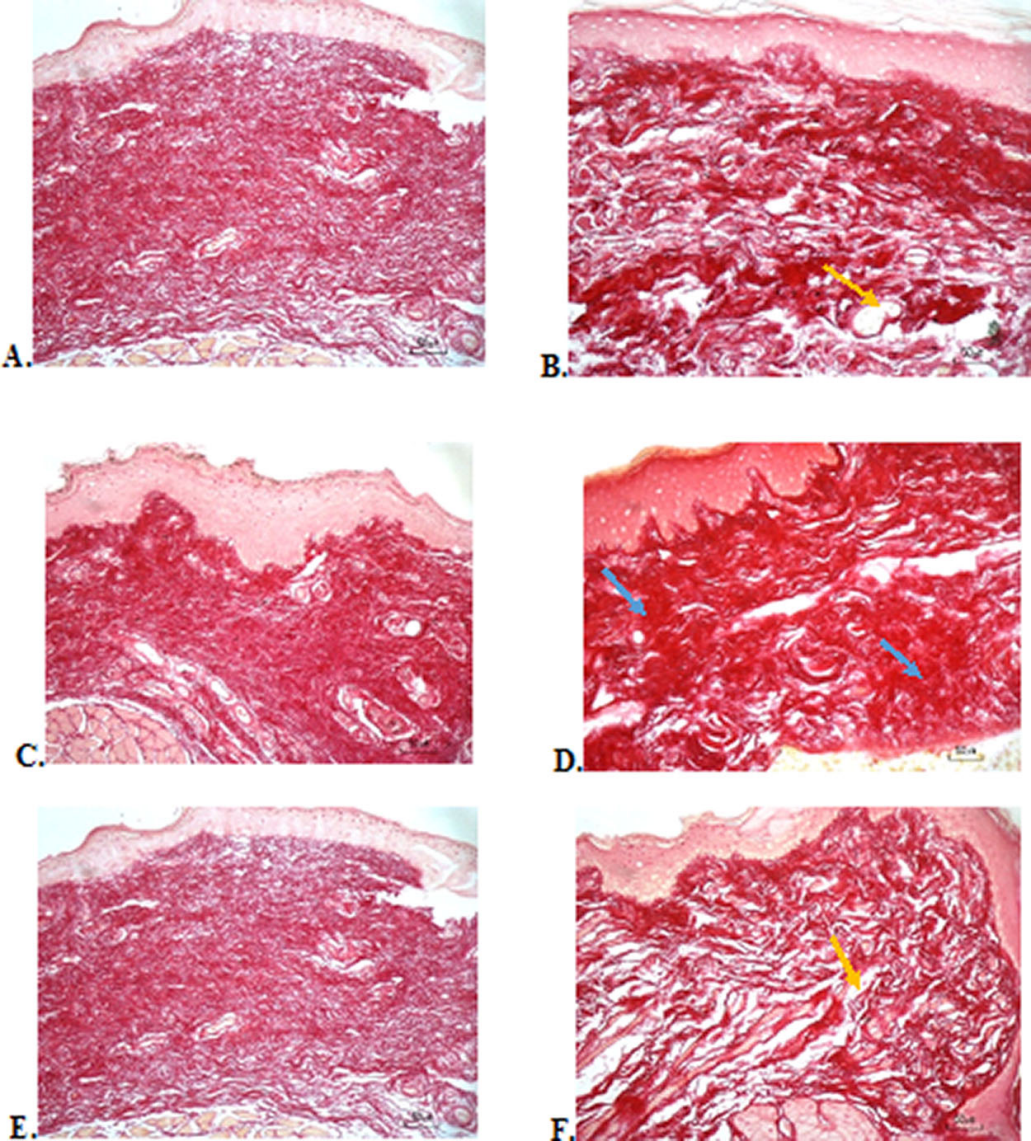
Collagenase analysis of the ulcerations in the buccal mucosa on the seventh day. (A) Negative control: base gel; (B) Positive control: triamcinolone acetonide in orabase; (C) F1: 20% areca nut:80% chrysanthemum; (D) F2: 50% areca nut:50% chrysanthemum; (E) F3: 80% areca nut:20% chrysanthemum; (F) Normal group: without ulcer and treatment (Picosirius red staining, 40×). Yellow arrow: Disintact collagen bundles. Blue arrow: Intact collagen bundles.

The results of red picrosirius staining on the last experimental day showed the highest collagen deposition in the F3 group with a mean of 25.4 ± 2.12, while collagen deposition in low level was seen in the F2, normal, and negative control group (12.63 ± 2.28, 9.04 ± 1.05, 5.63 ± 2.28, respectively) ( Table 6). Figure 4B (Positive control) and 4D (F2 group) showed regular, dense, and thick collagen, whereas, in the negative control ( Figure 4F), collagen deposition showed thin, irregular, and many empty gaps. The ANOVA analysis showed significant differences ( *p* < 0.05) in collagen expression between groups ( Table 6). The Tukey test demonstrated significant differences between F2 and F3 groups. Other significant differences were evaluated between F1, F2, F3, and negative control groups.

## Discussion

The preparation of the oral topical gel in this study using a CMC-Na-based gel. The gel form was chosen because it is easy to dry and provides a cool feeling on the mucosal surface. The gel formulation was carried out by trial-error by modifying the gelling agent. The aim of designing a stable oral topical gel is to deliver antioxidant properties effectively over its shelf life. Gel stability can be affected by environmental stress such as temperature and humidity. The decrease in the effect of the active gel ingredients can be observed from physical and chemical changes such as color, shape, texture, odor, and pH. The stability test in our research is a step in optimizing the preparation based on the selection of the appropriate gelling agent level so that the oral gel can be stable in various storage settings. The organoleptic characteristics of the optimum gel (F III) showed promising stability because there were no significant changes in color, odor, texture, and shape. The gel is said to be homogeneous if there is an even color equation and no different particles are found. The consistency, acidity, and spreadability of the gel are related to the comfort of use on the surface of the oral mucosa. The disadvantages of the gel are that it cannot adhere tightly to the mucosa surface and is easily soluble in saliva. This research showed that viscosity stability is an important consideration in the flow and texture properties of the gel. The stability of the constituents extract in the gel is also important because the pharmacological properties depend on the chemical viability of the extract. As pointed out above, the catechins are pharmacologically active constituents of the areca nut; however they are more stable at high concentrations around pH 4 and low temperature. ^9^ Their stability is affected by several factors such as pH, temperature, and concentration in solution state. ^10^

In the present study, we demonstrated that application of the oral gel to ulcerated lesions for 6 days showed significant lesion healing in the composition of the oral gel containing 50% areca nut:50% chrysanthemum and 80% areca nut:20% chrysanthemum. This significant wound healing was based on the clinical appearance such as ulcer size reduction, reepithelization over the ulcer base, and formulation of granulation tissue around the ulceration. These results have the same clinical appearance as the positive control group that using triamcinolone acetonide. Areca nut, as one of the main ingredients, contains phenolic and flavonoids which function as antioxidants. ^19^ Catechin is a component of flavonoids present in areca nut that acts as an antioxidant by chelating the ions and scavenging the free radicals particularly, superoxide (O ^2–^), peroxyl, hydroxyl radicals (·OH), and hence inhibit both DNA damage and lipid peroxidation, which can cause membrane damage. ^20^ Catechin can reduce the expression of interleukin-6 (IL-6) and IL-8 which function to overcome inflammation and increase the wound healing process by chemotactic for fibroblasts, accelerates their migration and stimulates deposition of tenascin, fibronectin, and collagen I during wound healing *in vivo.* ^21^ ^–^ ^24^ The success of using areca nut in oral gel to heal the ulcers is in line with a previous study using ointment with 2% ethanolic extract on burns on the skin. ^10^ This study also used a combination with chrysanthemum. Previous studies have shown that chrysanthemum has antioxidant, antiproliferative, antimicrobial, acetylcholine esterase inhibition, antitimelanogenic, and antiviral activities, especially *Chrysanthemum morifolium, Chrysanthemum indicum*, and *Chrysanthemum coronarium.* The active phytochemistry content of chrysanthemum is apigenin-7-O-glucoside. ^25^

This present study observed the body weight of rats before and after application. There are similarities between ulcer repair and weight gain. A significant increase in body weight in the F2 and positive control showed faster healing of ulcers, making it easier for rats to consume more food. The use of healthy rats aims to evaluate the effect of ulcers in the oral cavity purely based on the ability of rats to gain healing process without being affected by systemic diseases that can affect rat’s body weight. Areca nut is capable of repressing prostaglandin E _2_ (PGE _2_) and arachidonic acid-induced inflammation, thereby helping to accelerate ulcer healing. ^26^ The analgesic or antinociceptive activity of the areca nut is originated by inhibition of prostaglandin synthesis. The oral gel form was chosen to facilitate absorption in the oral mucosa and it has a rapid cool soothing effect, although the drawback is that the gel is easily soluble in saliva. Application twice a day in the morning and evening regularly showed a fairly good cicatrization process in both groups (F2 and positive control). The increase in body weight in the rat showed that in the last few days the diet was not disturbed by the size of the ulcer.

Histological examination with HE staining on day seven showed no significant difference between all treatment groups, normal, positive, and negative control groups. The oral gel containing a balanced composition of areca nut and chrysanthemum, negative control, and normal groups did not show any ulcers and the epithelial tissue had been completely remodeled. Meanwhile, the other group still showed fibrosis and inflammation without ulcers. Fibrosis, persistent neutrophil infiltration, and poor angiogenesis in the ulcer base are factors involved in the underlying mechanism of delayed healing ulcers. ^27^ The ulcer healing mechanism begins with ulcer base contraction which includes contraction of the ulcerated area, re-epithelization over the ulcer base, and formation of granulation tissue. The next step is epithelial regeneration and mucus secretion. This epidermal repair is regulated by a key growth factor (KGF) that is expressed at very high levels within 24 h of injury on dermal skin. ^28^ The buccal mucosa is a non-keratinocyte layer, so it is probably the most involved in the re-epithelization process in the oral mucosa is salivary mucin. Mucin is the primary gel-forming component of mucus that provide a critical layer of protection on the wet epithelial surface including gastrointestinal, female genital, and respiratory tracts. ^29^ Angiogenesis is the next step of ulcer healing. It allows the supply of nutrients and oxygen to the ulcerated area thereby giving the collagen in connective tissue and epithelium an opportunity to recover. Matrix formation is the last step of ulcer repair. This remodeling formation is regulated by the temporospatial expression of the fibrillar and basement membrane collagen (types I, III, IV), and matrix metalloproteinase-2 (MMP-2). Fibronectin is important for cell proliferation, adhesion, differentiation, and matrix formation.

In the present study, the analysis of TNF-α showed that the increase in the moderate expressions was showed by positive control and F2 groups. This is in line with research by Kobayashi *et al.* which showed that the water-soluble component in dried chrysanthemum has antioxidant ability to increase TNF-α. This activity was observed within 3 hours after ulcer formation. ^8^ TNF-α is a cytokine produced primarily by immune cells such as monocytes/macrophages, but several cell types, such as T and B lymphocytes, natural killer cells, neutrophils, fibroblasts, and osteoclasts can also secrete TNF-α in smaller quantities. ^30^ TNF-α levels which had the same score between F2 and positive control on the seventh day showed that the amount of this cytokine was still high detected in the local connective tissue. Prolonged or remaining elevated expression of TNF-α may occur for up to the third to the fifth day following the traumatic injury. ^31^ Prolonged increase in TNF-α especially in delayed healing reflects their augmented blood TNF-α, suggesting an association between impaired healing and hard work TNF-α in the inflammatory response so that wound healing is achieved. ^32^ In this study, we did not examine the level of TNF-α in the blood so that the role of areca nut-chrysanthemum oral gel to accelerate wound healing systemically cannot be explored.

Triamcinolone acetonide was chosen as a positive control because this preparation has efficacy as a synthetic corticosteroid that has anti-inflammatory, antipruritic, and anti-allergic effects. This preparation has the advantage of being an emollient dental paste that can attach the drug to the oral mucosal tissue so that it is not easily soluble in saliva. An important role of steroids in triamcinolone acetonide is to alter the localization of high junction proteins thereby increasing the adhesion density between epithelial cells. ^33^ A tight epithelial protective barrier can be achieved through tight cell adhesion. Contacts between adjacent cells are made up of tight junctions, adherens junctions, and desmosomes which help in preserving tissue homeostasis. This study showed that with the use of triamcinolone acetonide, F2, and F3 group on the seventh day, the ulcers had closed tightly without any inflammation. These results prove that there might be a possibility of areca nut–chrysanthemum oral gel also can close the adhesion bonds between epithelial cells so that the level of wound closure density which is similar to that of the group positive control (triamcinolone acetonide).

Collagenase analysis showed a lot of collagen deposition in the F3 group. The results of this study are in line with the fact that the alkaloids in areca nut are potent inducers to increase the amount of collagen. Previous research has shown that a series of synthetic arecaidine esters by oral mucosa fibroblast was closely correlated with the extent of collagen synthesis. ^34^ Alkaloids in the areca nut in a long term play a major role in the accumulation of collagen in oral submucous fibrosis (OSF). Collagen is a key component of the extracellular matrix that plays a critical role in the regulation of the phases of wound healing. Collagen exposure due to injury in the inflammatory phase will activate the clotting cascade, resulting in a fibrin clot that stops the initial bleeding. It also acts as potent chemoattractants for neutrophils, enhancing phagocytosis and immune response and modulating gene expressions. Collagen promotes anti-inflammatory, proangiogenic wound macrophage phenotype *via* microRNA signaling. In the angiogenesis phase, collagen stimulates angiogenesis *in vitro* and *in vivo* through the engagement of specific integrin receptors. The C-propeptide fragment of collagen I recruit endothelial cells, potentially triggering angiogenesis in the healing wound. ^35^

The results of the evaluation of collagen deposition after treatment, with picrosirius red staining, showed a picture of collagen with thicker density, large fiber size, and lots of collagen fibrils in the oral gel with higher composition of areca nut which consisted 80% of areca nut. This shows the good work of collagen to repair ulcers. Although the result of the collagenase assay has a significant *p* value, the range in the confidence interval (CI) is very wide. It indicates that these results have great uncertainty about the effect of the extract content in the gel for ulcer healing. ^11^ Further research is needed by using a larger sample size so that it can produce narrower estimated CIs and the estimated effect could become more reliable.

## Conclusion

The present study provides the activity of the healing effect of combination areca nut and chrysanthemum. This oral gel can improve ulcer wound healing through the role of TNF-α and collagen with the same results as topical steroid therapy.

## Data availability

### Underlying data

Dryad: Underlying data for ‘Evaluation of clinical, histology, TNF-α, and collagen expressions on oral ulcer in rats after treatment with areca nut and chrysanthemum oral gel’. https://doi.org/10.5061/dryad.8w9ghx3mw.

The project contains the following underlying data: body weight, oral ulcer’s size, histology and tnf-α score, collagen percentage.
