# Supplementary material for: Evaluation of clinical, histological, TNF-α, and collagen expressions on oral ulcer in rats after treatment with Areca catechu L. nut and Dendranthema grandiflora oral gel
Source: F1000Res. 2025 Sep 12;10:623. Originally published 2021 Jul 21. [Version 7] doi: 10.12688/f1000research.54887.7 (PMC12475897; doi:10.12688/f1000research.54887.7)
Supplement: Peer review history

# Reviewer report: Reviewer response for version 7

**DOI:** 10.5256/f1000research.187367.r413750

**Published:** 2025-09-26

**Authors:** Mingsan Miao

**Affiliations:** 1 Academy of Chinese Medical Sciences, Henan University of Chinese Medicine, Zhengzhou, China

**I have no further comments and recommend indexing.**

Is the work clearly and accurately presented and does it cite the current literature?

Yes

If applicable, is the statistical analysis and its interpretation appropriate?

I cannot comment. A qualified statistician is required.

Are all the source data underlying the results available to ensure full reproducibility?

Partly

Is the study design appropriate and is the work technically sound?

No

Are the conclusions drawn adequately supported by the results?

No

Are sufficient details of methods and analysis provided to allow replication by others?

No

Reviewer Expertise:

Pharmacology of Chinese Materia Medica

I confirm that I have read this submission and believe that I have an appropriate level of expertise to confirm that it is of an acceptable scientific standard.

# Reviewer report: Reviewer response for version 5

**DOI:** 10.5256/f1000research.141358.r159868

**Published:** 2023-01-26

**Authors:** Mingsan Miao

**Affiliations:** 1 Academy of Chinese Medical Sciences, Henan University of Chinese Medicine, Zhengzhou, China

1. For example, it is better to have a simple model group.

 2. It is better to provide the appraiser and Latin of betel nut and chrysanthemum for research; There should also be the extraction method of chrysanthemum.

 3. The weight range of rats is a little large, generally the same experiment does not exceed 20 grams.

 4. The level and certificate number of rats shall be provided; Provide the license number of rat animal center; Provide the source and batch number of main reagents and drugs.

 5. There are 12 in each group in the method, why is the result 18?

 6. Pathology is best to have semi-quantitative results; The basis for sample selection, extraction process and sample quantity in the process of immunohistochemistry should be supplemented.

 7. It is reported in the literature that long-term use of betel nut may cause oral cancer. Why should we choose betel nut to treat oral ulcer?

 8. The discussion of the experimental results can be divided into categories and appropriately compressed.

Is the work clearly and accurately presented and does it cite the current literature?

Yes

If applicable, is the statistical analysis and its interpretation appropriate?

I cannot comment. A qualified statistician is required.

Are all the source data underlying the results available to ensure full reproducibility?

Partly

Is the study design appropriate and is the work technically sound?

No

Are the conclusions drawn adequately supported by the results?

No

Are sufficient details of methods and analysis provided to allow replication by others?

No

Reviewer Expertise:

Pharmacology of Traditional Chinese Medicine

I confirm that I have read this submission and believe that I have an appropriate level of expertise to state that I do not consider it to be of an acceptable scientific standard, for reasons outlined above.

# Response

**Published:** 2025-08-27

**Authors:** Liza Sari

**Affiliations:** Dentistry Faculty Syiah Kuala Indonesia, Indonesia

**1. For example, it is better to have a simple model group.**

 We divided the test animals into six simple groups: positive control, negative control, normal (no treatment), and three treatment groups.

 These four groups are very important to determine whether the extract really works on the cells of the test animals. In this study, we also conducted tests to support each other's valid results.

 • In the first stage, we tested the best gel dosage based on pH, viscosity or consistency, spreadability, organoleptic characteristics, and homogeneity. This dosage will be used for treating the test animals.

 • In the second stage, we clinically observed the animals by weighing their body weight and measuring ulcer size before and after treatment.

 • In the third stage, we performed three microscopic examinations: histopathological staining, TNF-α immunostaining, and collagenase analysis.

 We strive to ensure that readers can understand the steps taken in this research to support the best possible results.

**2. It is better to provide the appraiser and Latin of betel nut and chrysanthemum for research; There should also be the extraction method of chrysanthemum.**

 We have changed all the term betel nut into *A. catechu*, and chrysanthemum into *D. Grandiflora.*

 We have added the maceration method in the “extract preparation section.”

**3. The weight range of rats is a little large, generally the same experiment does not exceed 20 grams.**

 We have corrected. In this study, we used Sprague Dawley rats from the Indonesian veterinary laboratory in Bogor, West Java, which generally have an average weight of 110-128 grams.

 Sprague Dawley rats were selected for this study because the ulcers that we need to form have a large diameter of 10 mm and a depth of ±2-3 mm. It will be created on their buccal mucosa. Ulcers formed in the buccal mucosa will require a wider buccal mucosa wall. Adult mice (weighing 20-40 grams) were not used in this study because we expected that using large rats, such as Sprague Dawley rats, would result in the formation of perfect ulcers and make their healing easier to observe.

**4. The level and certificate number of rats shall be provided; Provide the license number of rat animal center; Provide the source and batch number of main reagents and drugs.**

 Specific license numbers for test animals at the IPB University School of Veterinary Medicine and Biomedical Sciences are not publicly available, but their use is regulated by the Ethical Use of Test Animals regulations that must be followed by every research facility in Indonesia. **This research has passed ethical approval**.

 Test animal license numbers are part of an institution's internal data related to ethical and regulatory compliance. Specific information, such as license numbers, is not usually published for reasons of confidentiality and data security. All use of test animals in Indonesia must comply with the ethical guidelines and recommendations issued by the Test Animal Ethics Committee (KEPK).

 We have added the brand and reagent numbers to the manuscript:

 phosphate-buffered saline (PBS) (Sigm-Aldrich; P5493), anti-TNF-α antibody (RRID: AB_2892586; TNFA/1172) (ab220210Abcam), the secondary antibody goat anti-rabbit IgG H&L horseradish peroxidase (HRP) (RRID: AB_955447; ab6721-Abcam), Betazoid DAB chromogen solution (BDB2004H-Biocare Medicare LLC), Picro-Sirius red solution (Abcam; ab246832) (solution A), Adobe Photoshop CC 2017 (RRID:SCR_014199; GNU Image Manipulation Program (RRID:SCR_003182) is an open-access alternative) and ImageJ (v1.50i) (RRID:SCR_003070) software, SPSS 20 software (SPSS Inc., Chicago, USA) ( **RRID:SCR_019096**); JASP ( **RRID:SCR_015823**). ketamine HCl injection; OGB dexa, xylazine 20; Pantex Holland.

**5. There are 12 in each group in the method, why is the result 18?**

**Thank you very much for the correction. Please let me explain the difference between the 12 rats in each group (materials) and 18 specimens (in the results).**

 “Effect of *A. catechu* and *D. grandiflora* oral gel on histological scores, number of connective tissue cells that expressed TNF-α, and percentage of collagen deposition area on the 7th experimental day (n = **18 specimens**)”.

 We indeed used 12 rats in each group (6 groups). After completing the study, we took ulcer tissue (biopsy) after the healing process. In each group (containing 12 rats), we took 3 biopsy tissues representing the 12 rats in 1 group for microscopic analysis (HE staining, picosirius staining, and immunohistochemistry). Taking 3 specimens in each group was considered to represent the overall healing process. This was also due to funding limitations in the study.

 We hope reviewers can understand the limitations of this study.

**6. Pathology is best to have semi-quantitative results; The basis for sample selection, extraction process, and sample quantity in the process of immunohistochemistry should be supplemented.**

 Thank you for the correction. We have corrected as instructed. 

**7. It is reported in the literature that long-term use of betel nut may cause oral cancer. Why should we choose betel nut to treat oral ulcers?**

 Thank you for the detailed correction.

 Areca nut was chosen as a natural herbal ingredient because the quality of areca nuts in our region is very good. The percentage of patients with oral cancer is very low. Based on our survey of 87 cancer patients undergoing chemotherapy at our regional public hospital, only 4.87% suffered from oral squamous cell carcinoma with the risk factor of smoking tobacco with chewing betel nuts, not only areca nut. In addition, based on several references:

 Areca nuts themselves do not contain carcinogens, but the carcinogenic effect is caused by nitrosamines produced by the nitrosation process of alkaloids (arecoline) from dried areca nuts when chewed and digested in the stomach under acidic conditions over a long period of time and without control. ^1^ Combination with nitric oxide produced by bacteria causes the formation of methylnitrosaminopropionitrile, which has been proven to cause carcinogenesis in animal tests. ^2^ This endogenous nitrosation is significantly higher, especially in people with poor oral hygiene. ^2^ When areca nuts are mixed with tobacco and chewed by people with poor oral hygiene, a large amount of nitrosamine products accumulate. ^3^ This process occurs continuously over a long period of time because these nuts are addictive. Several studies have also reported an increase in reactive oxygen species, such as hydroxyl radicals in the oral cavity due to the combination of polyphenol autooxidation from areca nuts and high alkaline pH of lime. ^4^ When areca nuts are chewed together with betel leaves and lime, these two ingredients cause erosion of the mucous membrane, making it easier for carcinogens to penetrate cells through the mucosa. ^5^ Some communities in India and Pakistan use factory-packaged areca nuts called gutka and pan masala. Gutka consists of areca nuts, betel leaves, tobacco, and lime, while pan masala does not contain tobacco. Approximately 40% of gutka and pan masala packages are contaminated with carcinogenic aflatoxins from fungi of the species Aspergillus flavus, Aspergillus niger, and Rhizopus spp. ^6^

 References:

1. Bhisey RA, Boucher BJ, Hsi-Chen TH, Gajalakshmi V, Gupta PC, et al. Monographs on the Evaluation of Carcinogenic Risks to Humans.Betel-Quid and Areca-Nut Chewing and some Areca-Nut Related Nitrosamines. *IARCS Monographs* 2004;85:1-293.
2. Nagao T, Ikeda N, Warnakulasuriya S, Fukano H, Yuasa H, et al. Serum antioxidant micronutrients and the risk of oral leukoplakia among Japanese. *Oral Oncol* 2000;36:466-70.
3. Carossa S, Pera P, Doglio P, Lombardo S, Colagrande P, et al. Oral nitric oxide during plaque deposition. *Eur J Clin Invest* 2001;31:876-9.
4. Nair U, Bartsch H, Nair J. Alert for an epidemic of oral cancer due to use of the betel quid substitutes gutkha and pan masala: a review of agents and causative mechanisms. *Mutagenesis* 2004;19:251-62.
5. Johnson NW, Jayasekara P, dan Amarasinghe HK. Squamous cell carcinoma and precursor lesions of the oral cavity: epidemiology and aetiology. Periodontology 2000. Singapura: John Wiley and Sons; 2011.
6. Anttila A, Bhat RV, Bhond JA, Bhorgoff SJ, Bosch FX, et al. Monographs on the Evaluation of Carcinogenic Risks to Humans. Some Traditional Herbal Medicines, Some Mycotoxins, Naphthalene and Styrene. *IARCS Monographs* 2002;82:171-4.

**8. The discussion of the experimental results can be divided into categories and appropriately compressed.**

 Thank you. It is done.

# Reviewer report: Reviewer response for version 4

**DOI:** 10.5256/f1000research.139786.r153479

**Published:** 2022-11-09

**Authors:** Ali Akbar Nekooeian

**Affiliations:** 1 Cardiovascular Pharmacology Research Lab, Department of Pharmacology, School of Medicine, Shiraz University of Medical Sciences, Shiraz, Iran

Thank you for letting me to see this version of the manuscript.

 In the method section in a paragraph titled by experimental animal mentions that 25 rats were used. However, in two paragraphs after this the manuscript mentions that animals were divided in 5 groups of 12 each. Moreover, as I mentioned earlier the study suffers from the lack of a control group not subjected to oral abrasion and not receiving medication. This is very vital that can tell us if the model was working. Therefore, regrettably, I cannot recommend the publication of the manuscript.

Is the work clearly and accurately presented and does it cite the current literature?

Yes

If applicable, is the statistical analysis and its interpretation appropriate?

Partly

Are all the source data underlying the results available to ensure full reproducibility?

Partly

Is the study design appropriate and is the work technically sound?

Partly

Are the conclusions drawn adequately supported by the results?

Partly

Are sufficient details of methods and analysis provided to allow replication by others?

Yes

Reviewer Expertise:

NA

I confirm that I have read this submission and believe that I have an appropriate level of expertise to state that I do not consider it to be of an acceptable scientific standard, for reasons outlined above.

# Reviewer report: Reviewer response for version 2

**DOI:** 10.5256/f1000research.77568.r136024

**Published:** 2022-05-11

**Authors:** Ali Akbar Nekooeian

**Affiliations:** 1 Cardiovascular Pharmacology Research Lab, Department of Pharmacology, School of Medicine, Shiraz University of Medical Sciences, Shiraz, Iran

The manuscript examines the effects of areca and chrysanthemum oral gel on oral ulcers using clinical, histological and biomarkers criteria. Although nicely-written the manuscript suffers from a major problem and that is the lack of untreated group of rats with oral ulcer. The manuscript in the current form cannot answer the question that if untreated rats are left for the period used in this study, they would recover without treatment.

Is the work clearly and accurately presented and does it cite the current literature?

Yes

If applicable, is the statistical analysis and its interpretation appropriate?

Partly

Are all the source data underlying the results available to ensure full reproducibility?

Partly

Is the study design appropriate and is the work technically sound?

Partly

Are the conclusions drawn adequately supported by the results?

Partly

Are sufficient details of methods and analysis provided to allow replication by others?

Yes

Reviewer Expertise:

Cardiovascular Pharmacology

I confirm that I have read this submission and believe that I have an appropriate level of expertise to state that I do not consider it to be of an acceptable scientific standard, for reasons outlined above.

# Response

**Published:** 2022-10-12

**Authors:** Liza Sari

**Affiliations:** Dentistry Faculty Syiah Kuala Indonesia, Indonesia

We understand that wounds on the buccal mucosa of rats can heal on their own, but the results of our study prove that there is a difference in wound healing in the group that received treatment and the group without treatment. In addition to the results from clinical examinations, differences in healing speed were also supported by differences in test results for TNF-alpha levels and collagenase activity. The technique of applying the oral gel to the oral mucosa of rats is important in this research process because at the time of application the rats are not anesthetized, so there is a possibility that the gel is not maximally absorbed in the oral mucosa and most of it dissolves in saliva and is swallowed by rats. In this study, the oral gel was applied over the wound and held for 1 minute. The drug partitions dissolved into the mucous membrane and within a few minutes will reach equilibrium with the drug in solution in the oral cavity.

 One of the previous studies revealed the existence of the term pseudo-equilibrium which refers to the balance of a drug in solution and a drug in tissue in a relatively short contact time in the oral cavity before a dose of the drug is swallowed. **This pseudo equilibrium has an absorption half-life of about 0.87 minutes (52 seconds).** **It means that the oral mucosa absorbed the drug partition in the oral gel.**

 However, the effectiveness of the efficacy of the areca nut chrysanthemum gel can only be seen through clinical examination of the difference in cure rates between the treated groups with positive and negative controls. Although the results of the histology examination showed no significant difference, however, the results of the immunohistochemistry test showed an increase in TNF alpha expression which plays a role in the wound healing process as well as an increase in the work of collagen.

 Several factors that influence the fast or slow absorption of drugs on the mucosal surface are permeability and blood supply. The buccal mucosa is the part that has a high permeability making it easier for drugs to enter. The buccal mucosa has a thin layer of nonkeratinized epithelium consisting of the stratum basale, filamentous, and distendum, facilitating the absorption of the drug substance into the epithelium. In addition, the buccal mucosa lacks the intercellular lamellar bilayer structure found in the stratum corneum and is more permeable. Another factor that increases tissue permeability is the rich blood supply in the oral mucosa, which causes the absorption of drugs from the oral cavity into the mucosal tissue to be faster

# Reviewer report: Reviewer response for version 2

**DOI:** 10.5256/f1000research.77568.r97319

**Published:** 2021-10-26

**Authors:** Nurrul Shaqinah Nasaruddin

**Affiliations:** 1 Department of Craniofacial Diagnostics and Biosciences, Faculty of Dentistry, Universiti Kebangsaan Malaysia, Kuala Lumpur, Malaysia

The author has made the changes and amendments accordingly.

Is the work clearly and accurately presented and does it cite the current literature?

Yes

If applicable, is the statistical analysis and its interpretation appropriate?

Yes

Are all the source data underlying the results available to ensure full reproducibility?

Partly

Is the study design appropriate and is the work technically sound?

Partly

Are the conclusions drawn adequately supported by the results?

Yes

Are sufficient details of methods and analysis provided to allow replication by others?

Partly

Reviewer Expertise:

Pathology, histology

I confirm that I have read this submission and believe that I have an appropriate level of expertise to confirm that it is of an acceptable scientific standard.

# Response

**Published:** 2021-10-26

**Authors:** Liza Sari

**Affiliations:** Dentistry Faculty Syiah Kuala Indonesia, Indonesia

Thank you very much for your correction to our writing. 

 It improved the quality of the article.

# Reviewer report: Reviewer response for version 1

**DOI:** 10.5256/f1000research.58412.r90097

**Published:** 2021-09-24

**Authors:** Nurrul Shaqinah Nasaruddin

**Affiliations:** 1 Department of Craniofacial Diagnostics and Biosciences, Faculty of Dentistry, Universiti Kebangsaan Malaysia, Kuala Lumpur, Malaysia

1. The major concern of this manuscript is the technique of treatment process. While the rats are fully awake, it is very difficult to apply the topical on the ulcerated buccal mucosa. The rats will chew the probe and it is very difficult to visualize the lesions for monitoring.  Since the mucosal epithelium is highly dividing cells, how sure are you the healing occur due to treatment given? 
2. Among all inflammatory cytokines, why TNF-alpha was chosen? 
3. The contrast color for special staining picrosirius red should be yellow and red, and the red indicates positive for collagen. However, the pictures were lacked with the contrast and all the tissues appear homogenously red. 
4. The author should briefly explain the histology findings in the figure legend. 
5. The clinical evaluation results were contradicted with histology evidence, whereby the author mentioned that the ulcer is almost healed. but histologically, it showed complete healing with thick stratified epithelium. 

Is the work clearly and accurately presented and does it cite the current literature?

Yes

If applicable, is the statistical analysis and its interpretation appropriate?

Yes

Are all the source data underlying the results available to ensure full reproducibility?

Partly

Is the study design appropriate and is the work technically sound?

Partly

Are the conclusions drawn adequately supported by the results?

Yes

Are sufficient details of methods and analysis provided to allow replication by others?

Partly

Reviewer Expertise:

pathology, histology

I confirm that I have read this submission and believe that I have an appropriate level of expertise to confirm that it is of an acceptable scientific standard, however I have significant reservations, as outlined above.

# Response

**Published:** 2021-09-29

**Authors:** Liza Sari

**Affiliations:** Dentistry Faculty Syiah Kuala Indonesia, Indonesia

Dear Reviewer,

 I would like to respond to the comment's report:

**1.** ***The major concern of this manuscript is the technique of the treatment process. While the rats are fully awake, it is very difficult to apply the topical on the ulcerated buccal mucosa. The rats will chew the probe and it is very difficult to visualize the lesions for monitoring.  Since the mucosal epithelium is highly dividing cells, how sure are you the healing occur due to treatment given?***

**Answer**: In this study, we performed oral gel application on the buccal mucosa of the rat twice a day at 8 am and 8 pm. This application is by holding the rats, opening his mouth gently and slowly, then applying to the wound and holding it for about 20-30 seconds. After that, the mice were isolated briefly for 1 hour to avoid eating and drinking. We try our best to make the gel last well even if it's only for a few minutes, considering that this gel dissolves easily in saliva. We have acknowledged that this is one of the limitations of this study. We hope that with further research, we can make a formulation of topical oral gel in orabase so that it can last longer in the mouth. However, despite the brief application of the buccal mucosa, cellular changes can be seen differently on immunohistochemistry examination and collagenase assay after seven days of treatment.

 The extent of absorption of materials through the skin or oral mucosa depends on the surface area over which the substance is applied, the concentration of the substance administered, the lipid solubility of the material or vehicle, whether the skin surface is intact, the skin thickness at the site of application, the length of time that the material is in contact with the skin surface, and the degree of skin hydration and surface occlusion, in that covered and well-hydrated skin absorbs substances faster than does uncover or dry skin.

 The surface of the buccal mucosa is covered by non-keratinized stratified squamous epithelium. Keratinized mucosa is less permeable to the drugs as compared to the non-keratinized mucosa. This causes the buccal mucosa to more easily absorb the drug than the gingiva and palate. The turnover of the buccal epithelium which is due to differentiation and desquamation of the epithelium also accelerates the wound healing process happens faster. The turnover time for the buccal epithelium has been estimated at 14 days. Oral mucosa is well-hydrated skin that can absorb substances faster than dry skin.

 We have added this explanation in the “ **Discussion**” section.

***2. Among all inflammatory cytokines, why TNF-alpha was chosen?***

**Answer: **This research was initiated with lesions originating from simple injuries. We hope that the development of the results of this study can help the development of herbal extracts to accelerate the healing of oral diseases such as mucositis, RAS, and traumatic ulcers.

 The reason why we choose TNF-α is that TNF-α is a central regulator of inflammation. TNF-α is also a crucial cytokine in the establishment and maintenance of inflammation in multiple autoimmune diseases. 

 Although its precise role in wound healing is not fully understood, however, Ritsu et al. ( *Ritsu M, Kawakami K, Kanno E, et al. Critical role of tumor necrosis factor* *-α in the early process of wound healing in skin.* *J. Dermatol. Dermatol. Surg. 2017. Vol 21(1). 14-19*) reported that TNF-α is involved in the early process of wound healing. It is quickly released and initiates inflammation at wound tissues. The wound healing was accelerated on day 3 when mice were treated with bioactive TNF-α. Another previous study reported that impaired healing in animal models and age-related delayed healing of acute human wounds exhibit raised local and systemic levels of TNF-α that may parallel the pro-inflammatory phenotype. Despite previous studies showed varying results, there is an interest in observing the effect of TNF-α levels in ulcer healing after application of an oral topical gel.

**3. *The contrast color for special staining picrosirius red should be yellow and red, and the red indicates positive for collagen. However, the pictures lacked contrast and all the tissues appear homogenously red.***

 The resulting photos consist of two types; polarized and non-polarized light microscopy. The polarized colors of Picosirius red staining depend only on fiber thickness and packing, not on the composition of the specific collagen type within collagen bundles. Under polarized light, the oral mucosa sections seem to show collagen type 1 which showed thicker and denser fibers on the seventh day. We don't know why the red color was very generalized in the oral mucosa section although the Picosirius staining was done according to the standard procedure (as you can see in Figures 1a and b, the section was taken from one of the photos of positive control).

 We searched the reason from another source (Kiernan JA. Sirius red for collagen staining protocol. The University of Western Ontario, Canada). It was said that if we used the polarized light, sometimes we will lose "the yellow background" of picric acid staining. The loss of the yellow cytoplasmic background is probably caused by the dehydrating process.





 Figure 1.a. Polarized light microscopy (Positive control)

 Figure 1. b. Non-polarized light microscopy (Positive control)

**4. *The author should briefly explain the histology findings in the figure legend.***

 Thank you for the advice given. We have added the histology findings in the figure legend.

 5. ***The clinical evaluation results were contradicted histology evidence, whereby the author mentioned that the ulcer is almost healed. but histologically, it showed complete healing with thick stratified epithelium.***

 All measurements were performed by the same operator. We analyzed the ulcers directly and through photos. Histological examination results were analyzed several weeks after the visual examination was performed. At that time, some lesions showed the tip of the wound still have a light and thin ulcer while the other end had healed completely. The clinical examination data are the actual data based on our observations before seeing the histological results. The histological score showed that the group F1, F3, and positive control (Figure 2) had a score of 1. The normal, negative control and F2 showed a score of 0. We decided to perform the first data just the way they were without adjusting with the histological assessment.

# Reviewer report: Reviewer response for version 1

**DOI:** 10.5256/f1000research.58412.r91931

**Published:** 2021-08-16

**Authors:** Gunawan Indrayanto

**Affiliations:** 1 Faculty of Pharmacy, Surabaya University, Surabaya, Indonesia

Some important methods and data were not reported:

1. Scientific identification of the plant was missing.
2. Stability evaluation of the extract was missing.
3. No validation data of the biological assays were reported, without any validation, the validity of the data cannot be confirmed (Z factors, Selective Index, etc.)
4. No need to report the significance statistic evaluations, data should be presented ad Mean +/- Confidence interval; p values cannot be trusted.
5. Add Gandevia *et al. *(2021 ^1^) and related references.

Is the work clearly and accurately presented and does it cite the current literature?

Partly

If applicable, is the statistical analysis and its interpretation appropriate?

No

Are all the source data underlying the results available to ensure full reproducibility?

Partly

Is the study design appropriate and is the work technically sound?

No

Are the conclusions drawn adequately supported by the results?

No

Are sufficient details of methods and analysis provided to allow replication by others?

No

Reviewer Expertise:

Analytical Method Development and its Validation; Natural Product Chemistry, Herbal Drugs

I confirm that I have read this submission and believe that I have an appropriate level of expertise to state that I do not consider it to be of an acceptable scientific standard, for reasons outlined above.

## References

[1] : Replication: Do not trust your p‐value, be it small or large. *The Journal of Physiology* .2021;599(11) : 10.1113/JP281614 2989-2990 10.1113/JP281614 33963767

# Response

**Published:** 2021-09-08

**Authors:** Liza Sari

**Affiliations:** Dentistry Faculty Syiah Kuala Indonesia, Indonesia

- *Scientific identification of the plant was missing.*
- **Response:** This research is a series of research on the benefits of areca nut seeds that has been started in 2012. We have published several publications since 2016 using areca nut extract from the same source (Aceh Besar). We apologize for the negligence or forgetting to re-enter the scientific identification that has been done. We have re-entered the plant identification in the “Extract preparation” section in “Methods”. Thank you for your accuracy in reviewing.
- *Stability evaluation of the extract was missing.*
- **Response: **We have added the stability evaluation in the manuscript.
- *No validation data of the biological assays were reported, without any validation, the validity of the data cannot be confirmed (Z factors, Selective Index, etc.)*
- **Response: **We have included the validation data in the manuscript especially the selectivity index of areca nut and chrysanthemum. The studies of areca nut on oral squamous cell carcinoma cell lines (HSC-2 dan HSC-3 cell lines) and human dermal keratinocytes (HaCat cell lines) have been published in 2 previous journals:
  - Sari LM, Subita GP, Auerkari EI. Potential antioxidant and cytotoxic activities of areca nut ( *Areca catechu* Linn.) extract in human oral squamous cell carcinoma and keratinocyte cells. Asian J Pharm Clin Res 2017;10(10):286-91.
  - Sari LM, Subita GP, Auerkari EI. (2019). Areca nut extract demonstrated the apoptosis-inducing mechanism by increased caspase-3 activities on oral squamous cell carcinoma *F1000 Res, 7*(723), 1-34.
- *No need to report the significance statistic evaluations, data should be presented ad Mean +/- Confidence interval; p values cannot be trusted.*
- **Response: **Data have been changed to “Mean ± SD, 95% CI, *p-value*”. Several statements in “Discussion” dan “Results” has undergone changes regarding data interpretations.
- *Add Gandevia et al. (2021) and related references.*
- **Response: **We have added: Gandevia S. Replication: Do not trust your p-value, be it small or large. J Physiol 2021;599(11):2989-90.
